# *MiTiSegmenter*: Software for high throughput segmentation and meshing of microCT data in microtiter plate arrays

**DOI:** 10.1016/j.mex.2022.101849

**Published:** 2022-09-07

**Authors:** Kendrick Connah, Buckley Michael, Charlotte Brassey

**Affiliations:** a Department of Computing Maths and Digital Technologies, Manchester Metropolitan University, M1 5GD UK; b School of Natural Sciences, Manchester Institute of Biotechnology, University of Manchester, M1 7DN UK; c Department of Natural Sciences, Manchester Metropolitan University, M1 5GD UK

**Keywords:** Computed tomography, Bulk scanning, Digitisation, Museum archiving

## Abstract

Lab-based microCT is a powerful means of visualising the internal structure of physical specimens deployed across the physical sciences, engineering and the arts. As its popularity has grown, demand for bulk digitisation of multiple samples within a single scan has increased. High throughput workflows can increase sample sizes and reduce scan time, yet downstream segmentation and meshing remain a bottleneck. We present *MiTiSegmenter* as a new tool for the bulk archiving of valuable zooarchaeological and palaeontological remains. We foresee *MiTiSegmenter* as particularly useful when incorporated into workflows that ultimately require the destructive testing of specimens, including sampling for ancient DNA and proteomics. The software may also play an important role in national museums' ongoing mass digitisation efforts, facilitating the high-speed archiving of specimen 3D morphology across extensive collections with very minimal user intervention or prior training.

• We present *MiTiSegmenter*, a software package for semi-automated image processing and segmentation of array-based batch microCT data.
• Implemented in Python, *MiTiSegmenter* expedites cropping, meshing and exporting samples within stacked microtiter plates, facilitating the rapid digitisation of hundreds-thousands of samples per scan.
• We illustrate *MiTiSegmenter's* capabilities when applied to bulk archiving of valuable zooarchaeological and palaeontological remains

## Graphical abstract

**Figure:**
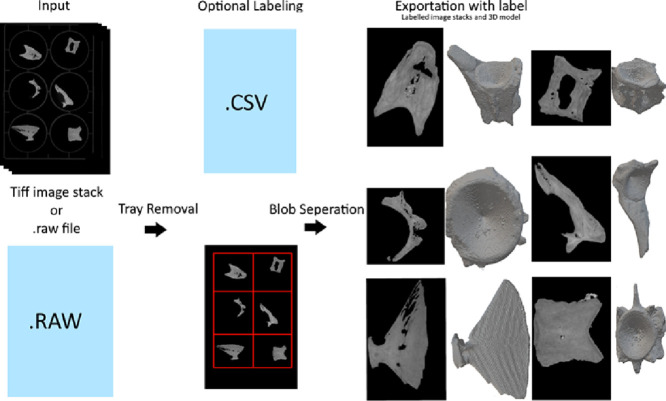

Specifications table

**Table:** 

Subject Area:	Agricultural and Biological Sciences
More specific subject area:	Zooarchaeology; Palaeontology; Museum Studies
Method name:	*‘MiTiSegmenter’* (MicroTiter Plate Segmenter)
Name and reference of original method:	NA
Resource availability:	Software executable, user manual, and example scans are available on Github: https://github.com/connahKendrickMMU/MiTiSegmenter Example Dataset: https://figshare.com/articles/Example_data_for_MitiSegmenter_software/12349847 Labels: https://figshare.com/articles/dataset/Example_labelling_spreadsheets_to_accompany_MiTiSegmenter/17899916

## Background

Broader access, falling costs and simplified protocols have ensured that 3D digitisation has become an essential component of many researchers’ workflow. Although powerful, digitisation can be a time-consuming endeavour, and there is an understandable desire to both reduce scan time and increase sample sizes whilst balancing any accompanying trade-off with scan resolution.

Bulk scanning (in which numerous discrete samples are digitised within a single scan ‘volume’) may be deployed to increase the throughput of a digitisation workflow significantly. Bulk CT scanning may take the form of multiple specimens arranged in a low-density mounting medium, scanned and manually segmented into individual components (rat foetuses [1]; corn kernels [2]). In some instances, this is accompanied by custom-written code to automate segmentation and downstream analyses (sorghum stems [3]). Most promisingly, such automated segmentation code has occasionally been deployed in combination with specially designed ‘holders’ or ‘cassettes’ to speed up further the digitisation process (timber cores [4]; rat hind limbs [5]).

**Fig. 1:**
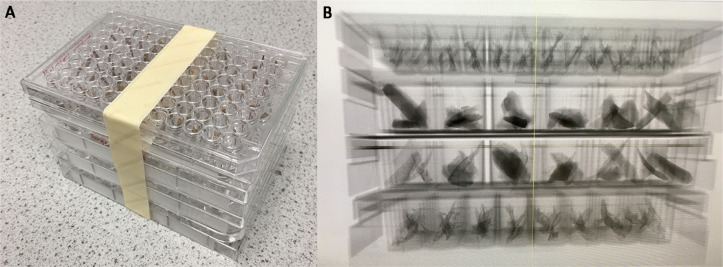
*MiTiSegmenter* software is used to automatically segment and export individual tomographic datasets from array-based bulk microCT scans. A. Four microtiter plates of varying well number (24, 48 and 96 wells) are used to mount 192 discrete skeletal specimens and are stacked in the z-direction prior to microCT scanning. B. A radiographic projection of the microtiter plate array.

No user-friendly GUI-based software exists for the explicit purpose of segmenting such bulk CT data whilst also retaining specimen IDs assigned externally to the software, such as museum labels or laboratory codes. We present *MiTiSegmenter*, a software package for the semi-automated processing and segmentation of array-based microCT data. Samples are arranged in a grid-like fashion within microtiter plates (Fig. 1), common biomolecular laboratory consumables, comprising flat plastic plates holding numerous ‘wells’. Microtiter plates are manufactured in multiple array dimensions, most commonly 6 × 4, 12 × 8 and 24 × 16 ‘well’ arrays. In guidance set out by the Society for Biomolecular Screening, industry standards are now in place for the footprint dimensions (ANSI SLAS 1-2004) and well positions (ANSI SLAS 4-2004) of 96, 384 and 1536 well density formats. Therefore, our proposed sample holders are low cost, standardised, scalable, stackable, and widely available to labs globally.

*MiTiSegmenter* is an important contribution to the field of microCT imaging, incorporating both an interactive GUI and intuitive specimen mounting protocol. Its novelty lies in using microtiter plates as sample holders, which may subsequently be stacked vertically, typically resulting in 3 or 4 plates per scan volume and sample sizes in orders of hundred-thousands of discrete specimens. Within the *MiTiSegmenter* software, scans are temporarily downsampled upon import to reduce processing times. Samples are segmented from the microtiter plates using Cel-shading and standard thresholding. Furthermore, individual specimens are isolated using 3D image blobbing. Due to the predictable grid-based nature of the microtiter plates, associated museum/laboratory ID codes may be imported from externally-generated spreadsheets alongside image data and incorporated into the workflow. Identification labels are assigned to each sample using the superimposed grid and reference spreadsheets. Finally, resulting image masks are then used as a guide for the export of the original high-resolution data as individual 8-bit tiff stacks and an accompanying .ply surface mesh, all whilst retaining original specimen IDs. This removes the need to assign file names to potentially hundreds-thousands of files manually, and crucially, reduces the potential for any naming transcription errors.

We provided a specific example of the incorporation of *MiTiSegmenter* into the workflow of Zooarchaeology by Mass Spectrometry (ZooMS; [6]). Archaeological bone assemblages comprise highly fragmentary skeletal remains. Recent applications of proteomic methods facilitate the identification of bone fragments to species-level using collagen-based peptide mass fingerprinting by MALDI-TOF Mass Spectrometry [7]. Yet this analysis is destructive, and valuable morphological data is lost when fragments are destroyed during collagen extraction. We highlight the value of *MiTiSegmenter* for the collection of bulk high-resolution microCT data from bone fragments prior to destructive sampling.

## Methodological requirement

There are several functional requirements that any high throughput CT workflow must meet:

*Optimise resolution -* Any mounting ‘holder’ must optimise the spatial density of samples within a given scan volume. In microCT, the x-ray cone beam ensures straightforward geometric magnification of the scanned object. Thus, the closer specimens may be positioned to the x-ray source, the higher the magnification on the detector panel and hence the smaller the voxel size [8]. Any holder must place multiple discrete specimens within as small a 3D volume as possible to reduce the maximum dimension of the real object.

*Enhance contrast -* Resulting CT datasets represent 3D maps of x-ray attenuation through a sample, which is itself a function of elemental composition and material density. Similar greyscale values will represent objects characterised by similar x-ray attenuation in the resulting CT dataset. To facilitate rapid, automated/semi-automated segmentation of samples away from the mount, any holder should differ in material properties from the specimens of interest to minimise overlap within the greyscale histogram. This is most often achieved through the use of low-density mounting materials.

*Efficiency -* CT datasets are often very large in size. A medical CT dataset might typically span 100MB-10GB, whilst a reconstructed lab-based microCT dataset will frequently occupy 10GB-100GB, depending upon detector size and bit depth (i.e. 32, 16 or 8 bit). Software for downstream segmentation of bulk CT scans must be capable of loading and processing such datasets in a timely manner.

*Traceability -* Samples within a single bulk CT dataset may originate from different species, strains or contrasting experimental protocols arranged in a documented array. When processing bulk CT scans comprising potentially hundreds-thousands of individual samples, the ability to reliably isolate, label and extract discrete specimens whilst retaining this valuable ID information is therefore essential.

*Flexibility -* To be broadly adopted by the academic community, any segmentation software must have the capability of functioning across a diverse array of sample holders and/or operate upon standardised mounts that are widely available to users.

### Software description

**Fig. 2:**
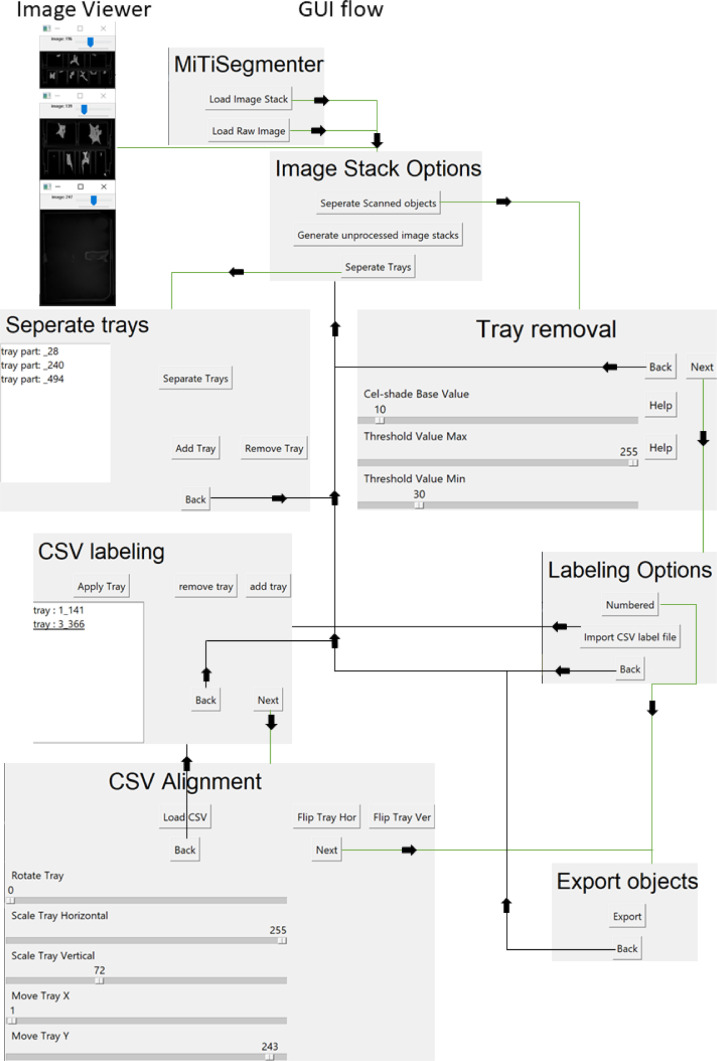
*MiTiSegmenter* GUI interface guides the user through each step of the process, providing real-time feedback on the user's choices. The simple GUI changes to the next/ previous window on the ‘next’ and ‘back’ button press. Thus, the user is not presented with a single multipurpose window and a potentially overwhelming number of functions and options upon first viewing. Each of the image viewer windows is resizable and floats separately, allowing users to reposition and resize as required. Not all changes affect the system's data, allowing users to go back and adjust until they are satisfied with the final result.

*MiTiSegmenter* is a GUI-based application released under the MIT license. It is built under Python 3.6 using the Tkinter GUI library. A path through the system is illustrated in Fig. 2.

**Fig. 3:**
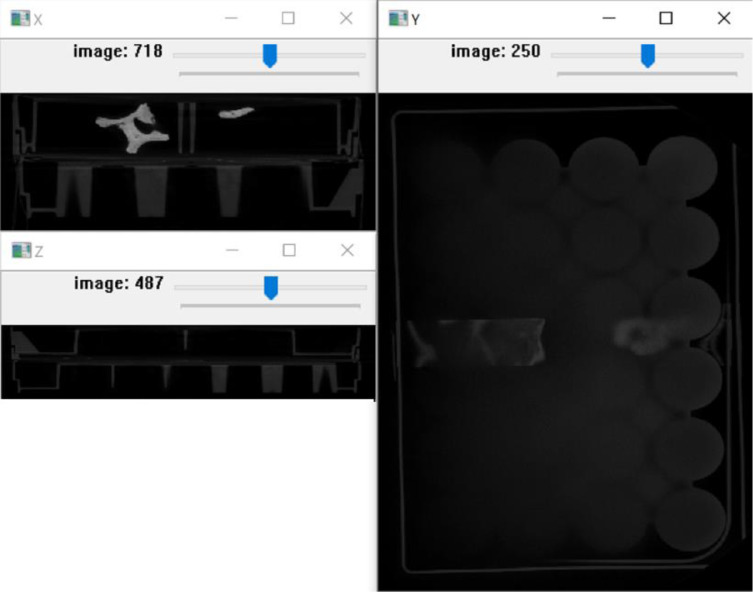
An example of the 3 floating windows opened by MiTiSegmenter on the example data. The views show an X, Y and Z slice view of the image stack.

A file menu loads and exports images and 3D models. *MiTiSegmenter* offers three orthogonal views of the dataset (Fig. 3) navigated using scroll bars (top).

## Image import

MicroCT stacks are very large, often exceeding 4K resolution. Large slice numbers (∼2000-3000) make storing frames into memory challenging. *MiTiSegmenter* requires either a .raw image or a folder of tiffs of any standard bit depth (6, 18, 32, 64). Data is downsampled upon loading to act as a template before the final protocol is applied to the original dataset, saving processing power and time by overworking end-to-end with full-resolution images. The user selects a downsample rate on importing the data. The user also can work end-to-end with the full dataset (reading multiple stacked ‘plates’ into memory at once) or pre-processing the data to isolate discrete plates and save them as separate tiff stacks first (saving on memory by reading a single plate at a time). As the images contain no scale information, *MiTiSegmenter* refers to a text file within the image folder for scale and sequence information. This file is typically generated at the reconstruction phase (with the extension ‘.info’) but can be generated internally if absent.

### Segmentation

In the most basic approach to segmentation, a global threshold (**Error! Reference source not found.**) assigns all pixels greater than or equal to a predetermined greyscale value to the foreground and the remainder to 0:

(1) $$X=\left\{ \begin{array}{c} 0,\;X<T_{lower}\\ X,\;X\geq T_{lower} \end{array}\right.$$

(2) $$X=\left\{ \begin{array}{c} 0,\;X>T_{upper}\\ X,\;X\leq T_{upper} \end{array}\right.$$

Where X is the pixel value, and T the threshold value, this assumes that all samples of interest will be denser (i.e. higher pixel value) than the surrounding mounting medium. As this is frequently not the case, as an initial approach, we employ greyscale bracketing Eq. (1) and ((2) deployed together), assigning all pixels outside a user-determined range to the foreground.

**Fig. 4:**
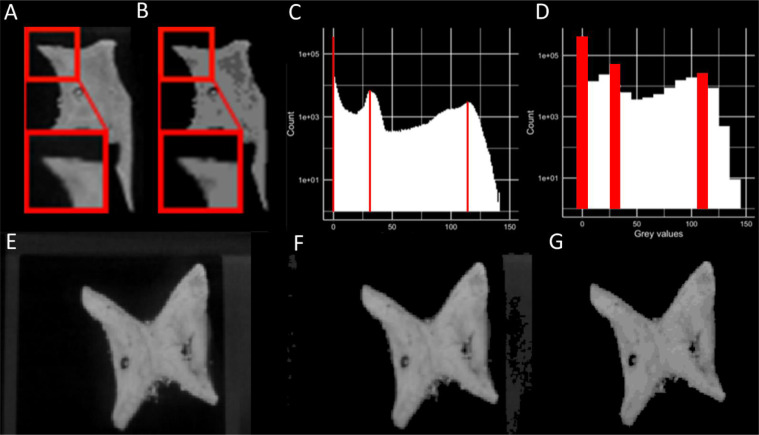
Thresholding implemented in *MiTiSegmenter*. A. The raw input data, with a partial volume ‘halo’ around the specimen edges; B. Cel-shading applied to group greyscale values, allowing for a more straightforward selection of threshold values; C. Original greyscale histogram of a single image. Peaks, highlighted red, at 0, 31 and 114 represent air, mount and sample, respectively; D. Histogram of the same data following Cel-shading with a base=10. E. Raw data with mount visible; F. Same data as E with global threshold applied; G. Same data as E with Cel-shading followed by thresholding applied.

CT images experience ‘partial volume averaging’ however; when two materials of different x-ray absorption occur within the same voxel, resulting greyscale values reflect their average. A ‘halo’ of lower greyscale voxels around object margins can result. Basic global thresholding may remove said intermediate voxels and erode object surfaces (Fig. 4A-B).

Furthermore, although mounting media is selected for minimal overlap with sample density, additional noise and scan artefacts can result in sections of the mount remaining within the threshold. Increasing x-ray projections or frame averaging can reduce noise but considerably lengthen scan time. For high throughput protocols, such methods may be undesirable, and some noise is likely inevitable.

An optional step is added to account for noise: namely *Cel-shading*. Cel-shading ‘ quantises’ the image gradient (i.e. reduces the number of greyscale values by binning similar values by a predefined factor, see Fig. 4C-D), applied through 3:

(3) $$X_{new}=X-\left(X\;\%\;C\right)$$

Where X is the voxel's intensity value and C the base value, for 8-bit images comprising 255 greyscale values, a base value of 10 produces Cel-shaded images of 25 grey values. Voxels are rounded upwards/downwards into adjacent bins, potentially adding or removing material from the mask. Binning simplifies the selection of an appropriate cut-off threshold and assists in noise removal Fig. 4C-D). It may, however, cause erosion at sample margins and must be used cautiously. After Cel-shading, the image is thresholded as per Eq. (1) and ((2) via a user-inputted threshold.

## Labelling

**Fig. 5:**
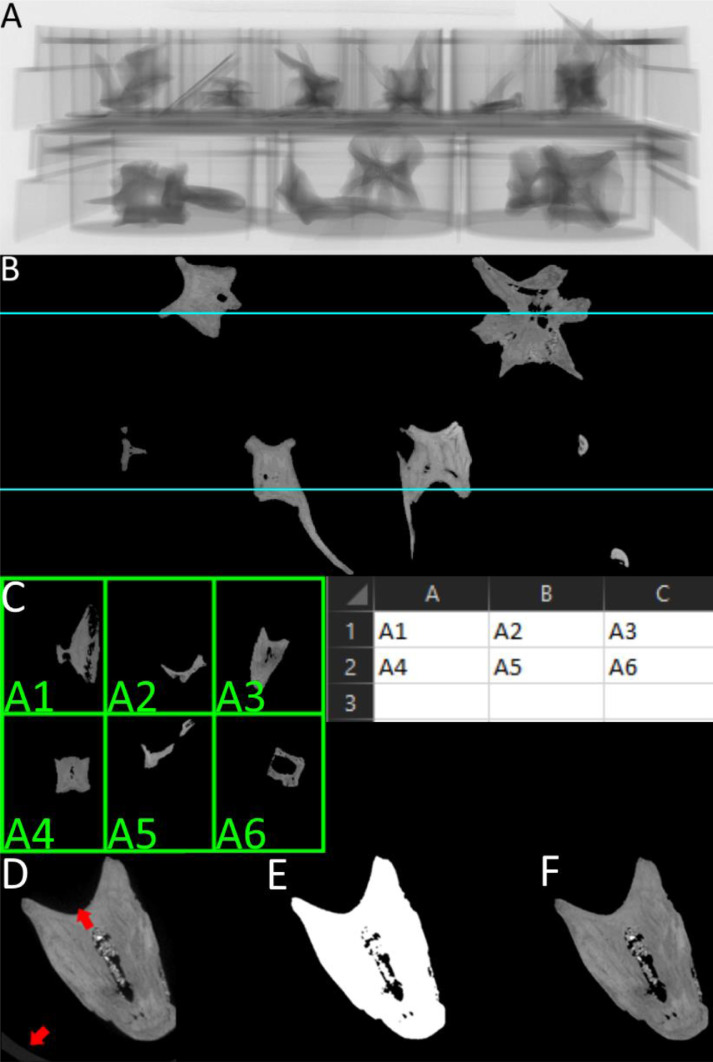
Stages of MiTiSegmenter. A, An radiograph of the stack. B, An example of a thresholded 2 plate stack where the blue line represent the midway point of each tray. C, an example of a labelling grid imported from a CSV file, for visualation the labels are shown in the grid, but in the program only the four corners are labeled. D, a visualisation of raw image, where the microtiter plate and scanner noise is visible. Where the bottom red arrow is the tray, and the top arrow is a edge blur create by the scanner difficult to see in most images. E, an example of a segmentation binary mask, produced that removes the blurred edges and tray. F, The processed image containing only the sample, but preserving internal detail.

Most samples undergoing high throughput microCT possess preassigned specimen numbers (e.g. museum accession numbers, trial IDs, site codes). As *MiTiSegmenter* permits the digitisation of hundreds or even thousands of samples simultaneously, assigning specimen numbers to exported data is essential. Thus, the semi-automatic labelling of outputted 3D models and image stacks is enabled. *MiTiSegmenter's* labelling method relies upon evenly spaced microplate arrays. *MiTiSegmenter* requires specimen numbers to be recorded in an accompanying .csv spreadsheet in a grid format reflecting the underlying plate layout for the auto-labeling. However, the specimens can be exported numbered, which does not require a csv file. An example scan (https://figshare.com/articles/dataset/Example_data_for_MitiSegmenter_software/12349847) and labelling spreadsheets (https://figshare.com/articles/dataset/Example_labelling_spreadsheets_to_accompany_MiTiSegmenter/17899916) are provided on Figshare. The system will highlight the IDs of corner samples, to allow the user to manually verify the scan is not mirrored. However, for bulk scanning items that do not require an ID, the system will assign a number to each separate object. The number of plates stacked vertically is detected by identifying consistent gaps in the z-direction between samples, determined by the presence/absence of thresholded samples (when a layer has a pixel count less than the min blob size). A plate's centre in the z-direction is determined at the median slice between the start and end slice of said plate. .csv files for the relevant plates are imported from a dialogue box, and labelling grids of corresponding dimensions are automatically generated over the data (Fig. 5C). The grid may be translated, rotated, flipped and scaled by the user to ensure agreement between labels and underlying data. This allows the user to align the grid with the specimens in the scan. The centroid of each grid square is matched to the centroid of the nearest masked sample via Euclidean distance, linking specimens and the labelling spreadsheet, this allows for specimens to not entirely be in the grid. In addition, the program can handle empty cells, as well as when to samples are in the same well, e.g., damage during transfer. File names are assigned at export on this basis.

Stack export

*MiTiSegmenter* exports individual 8-bit TIFF stacks for each specimen and a corresponding 3D mesh. Although initially downsampled to preserve memory, *MiTiSegmenter* refers back to the original files and exports full-resolution image stacks. Users are presented with three export options:

1. ‘Raw unprocessed images’. Cropped raw input data without further processing, potentially including sections of the mount (Fig. 5D).
2. ‘Segmentation mask’. A binary mask representing the specimen detected during imaging blobbing (Fig. 5E).
3. ‘Processed’. The result of segmentation. Pixels from the original dataset falling within the binary mask are segmented (Fig. 5F).

### 3D model export

*MiTiSegmenter* also exports individual surface meshes. To preserve as much of the original data as possible, smoothing is not currently performed during mesh generation. Users may further process resulting meshes depending upon their requirements.

**Fig. 6:**
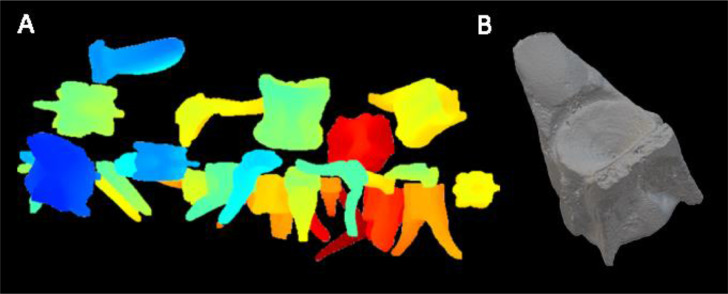
A. Point cloud rendering of three stacked microtiter plates containing archaeological specimens. Colours reflect the distance of the points from the origin (0, 0, 0); B. 3D surface rendering of an archaeological skipjack tuna vertebra (20 mm x 18 mm x 12 mm) microCT scanned at a resolution of 48 microns.

Once samples are identified using blobbing, each blob is cropped from the original dataset, resulting in a 3D matrix comprising a stack of 2D images (Fig. 5F). Voxels within the mask (value=1) are assigned as points within the 3D point cloud (Fig. 6A). To reduce memory and processing requirements, users may opt for canny edge detection [11], making resulting point clouds “hollow” rather than filled (models retain internal surfaces and void spaces).

From point clouds, *MiTiSegmenter* generates 3D meshes via the Marching Cube algorithm [9,10] and exports in ‘.ply.’ format readable by most 3D visualisation programs and 3D printers. As the models are offset in their world coordinates due to originating from a larger original matrix, models are re-centred around their origin (0,0,0). Finally, the voxel size of the scan (as provided in the accompanying text file or user input) converts the scale of the mesh from arbitrary integers into real units. A rendering of a 3D mesh is shown in Fig. 6B.

## Method validation

MicroCT data was collected at the Manchester X-Ray Imaging Facility (MXIF), the University of Manchester, using a Nikon XTEK XTH 225kV cabinet scanner (Nikon Metrology, Tring) equipped with a 3000 × 3000-array detector and tungsten target. The stacked specimens were scanned at a resolution of 48 microns, a voltage of 120 kV and current of 110 uA, using a 0.1 mm copper filter and 5013 projections. Raw projection data were reconstructed into a 3D volume in Nikon's XCT software and export as a 32-bit .vol file of ∼80GB. An example 8-bit raw dataset is available for download from Figshare (https://figshare.com/articles/dataset/Example_data_for_MitiSegmenter_software/12349847), downsampled to 95 microns to reduce file sizes (∼670MB) and facilitate sharing.

Load time into *MiTiSegmenter* was ∼4 minutes for a 3191*3191*1869 voxel 8-bit dataset (PC hardware: Intel i7-7700 Quad core 3.60GHZ, 64GB DDR4/2400mhz, GTX 1080 Ti (11GB)), downsampled by a factor of 4. For Cel-shading, we used a base value of 6 as this successfully grouped most of the microplate voxels into a single histogram bin and a threshold of 52 to remove background noise. Image blobbing removed any remaining voxels of the mount, falling below that of the smallest sample. Having assigned the labelling grid, export time for the three TIFF stacks (raw, mask and processed) plus .ply surface models averaged 40 seconds per specimen.

**Fig. 7:**
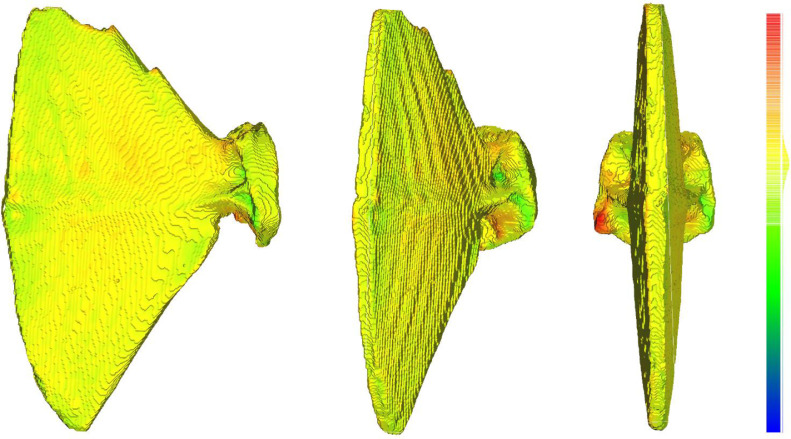
Comparision of mesh topology. Manually segmented InVesalius mesh was aligned to the MiTiSegmenter mesh in CloudCompare using Iterative Closest Point. Colours represent calculated distances between the two meshes, superimposed back onto the MiTiSegmenter mesh. Hot colours represent greatest deviation between meshes. Mean intermesh distance was calculated as 7.7 microns (standard deviation of 120 microns) indicating excellent agreement between meshes considering the scan resolution of 48 microns. One parhypurapophysis (far right image, red region) failed to be captured adequately by the global threshold of MiTiSegmenter, but was manually resolved ‘by eye’ during segmentation in InVesalius. Specimen is approximately 30mm in maximum dimension.

To compare the output of *MiTiSegmenter* to those derived from a more user-intensive ‘manual’ CT segmentation workflow, the same dataset was imported into the software ‘InVesalius’ [14]. InVesalius is a popular piece of open-source software for reconstructing CT and MRI images. Still, it lacks functionality for the automated file naming and export of multiple cropped ROIs and surfaces, as available in *MiTiSegmenter.* To simulate the manual approach to CT segmentation often applied to archaeological and palaeontological samples, a single specimen was selected from the scan and a ‘best effort’ segmentation by one of the authors (CAB). This was conducted blind to the segmentation within *MiTiSegmenter* detailed above. An initial global greyscale threshold of 50 was applied across the scan, as determined ‘by eye’ to best differentiate bone from air without any discernable haloing around the specimen. The individual specimen of interest was manually selected by clicking on the sample of interest using the ‘select parts’ function and moved to a new mask. Subsequently, a threshold paintbrush tool (greyscale value 40-255) was applied slice-by-slice to manually capture additional specimen regions characterised by a slightly lower material density and missed by the global threshold. A 3D surface model was created using the ‘binary’ function, avoiding any smoothing and thus being directly comparable to the surface model produced by *MiTiSegmenter,* and exported as a .ply file. Meshes exported from *MiTiSegmenter* and InVesalius were compared using mesh distances calculated in CloudCompare [15]. Fig. 7

The *MiTiSegmenter* workflow outlined above results in the segmentation, labelling and surface meshing of ∼200 discrete samples from microCT data. Elsewhere, the ‘Separate Objects’ module of Avizo, for example, already allows for separating discrete ‘blobs’ from a binarised dataset and assignment of a unique index label prior to meshing. Yet as far as the authors are aware, no comparable software exists for the automated file naming of exported data based on an associated data frame. Such functionality depends on the grid-like layout of samples associated with the use of microtiter plates. It highlights the benefit of *MiTiSegmenter* when deployed on extensive collections within a museum setting, in which the maintenances of specimen IDs are essential throughout the digitisation process. Further downstream analyses such as density, porosity or fibre orientation calculations, and geometric morphometric analyses, may be conducted on the exported image stacks and surface meshes.

### Future directions

Here we have introduced *MiTiSegmenter*, a Python-based software application for the high throughput segmentation, labelling, meshing and export of bulk microCT data of specimens mounted in microtiter plates.

In the future, we foresee the incorporation of several additional features:

- Microtiter plates are an essential component of the proteomic workflow for which *MiTiSegmenter* was initially designed. Yet using such rectangular plates demands a wider field of view in microCT than may otherwise be necessary, resulting in a compromise with scan resolution. A cylindrical mounting arrangement would instead be optimal. For those wishing to achieve higher resolution scans *without* using microtiter plates (e.g. researchers beyond the field of proteomics), we foresee providing .stl files for 3D printing cylindric mounting disks, accompanied by a modified labelling workflow in *MiTiSegmenter.*
- *MiTiSegmenter* currently uses a very simplistic segmentation protocol, which may struggle with multiphase specimens, or those overlapping considerably in density with the mounting medium. Given the large volume of material available, a trainable machine-learning approach to segmentation may be further explored, as it has already been successfully deployed on microCT data elsewhere [12,13], or advance thresholding technique.
- Although conservative, the use of the Marching Cubes algorithm generates surface meshes of a blocky texture. In future versions, users may be provided with a suite of meshing options including surface smoothing, which may be preferable for 3D visualisation or 3D printing purposes.
- A comparison of advanced threshold and segmentation techniques, such as Otsu [16] for thresholding and Deep learning threshold techniques to segment [17], the tray, objects and tray voids. This is to remove the Human variance and error, in the process and allow for a more intuitive approach to the system.

## Data Availability

Data will be made available on request.
